# Analysis of the relationship between antidepressants and seizures based on the food and drug administration's adverse event reporting system database

**DOI:** 10.1016/j.clinsp.2025.100727

**Published:** 2025-07-15

**Authors:** Dan Zou, Qiaozhi Hu, Lei Yu, Bin Wu

**Affiliations:** Department of Pharmacy, West China Hospital, Sichuan University, Chengdu, PR China

**Keywords:** FAERS, Antidepressants, Seizures, Adverse events, Drug safety

## Abstract

**Background:**

Antidepressants, a cornerstone of depression treatment, are increasingly scrutinized for their safety concerns. The seizure risk of antidepressants has garnered attention. However, the current research on the relationship between antidepressant use and seizures remains unclear, prompting this study to investigate potential associations using data from the FAERS database.

**Aim:**

This study sought to assess the correlation between seizures and different antidepressants in individuals without pre-existing epilepsy or neurological comorbidities, as well as to explore potential variations in the occurrence of adverse events linked to each specific antidepressant drug.

**Methods:**

A disproportionality analysis was conducted to identify potential risk indicators within the FAERS database. Signals were detected using both the Reporting Odds Ratio (ROR) via the frequency approach and the Information Component (IC) through the Bayesian approach.

**Results:**

A total of 15,940,383 FAERS reports from January 1, 2004, to December 31, 2023, were analyzed. 14 antidepressant drugs were associated with seizures, as defined by 41 preferred terms.

**Conclusion:**

This study identified 14 antidepressant drugs that showed significant associations with seizures. Bupropion had the highest seizure ROR among the antidepressant drugs evaluated in this study.

## Highlights

• 14 antidepressants show a significant seizure risk association in the FAERS analysis.
• Bupropion shows the strongest seizure signal with the highest reporting odds ratio.
• TCAs/tetracyclics elevate seizure risk, particularly clomipramine and maprotiline.
• SSRIs/SNRIs require safety reconsideration as seizure risk persists.
• Mirtazapine is linked to seizure risk despite low initial reports.

## Introduction

Depressive disorders are highly prevalent in the general population and significantly impact quality of life. Consequently, there has been a marked increase in antidepressant utilization globally, particularly with the expanding indications for these medications beyond major depressive disorder to include anxiety disorders, chronic pain syndromes, and other psychiatric conditions^1^. This upward trend in prescription rates underscores the need for heightened vigilance regarding their potential seizure effects. Recent research indicates that certain antidepressant medications modulate neurotransmitter systems, particularly those associated with serotonin and norepinephrine^2^^,^^3^. These medications may impact the excitatory and inhibitory functions of neurons, potentially inducing abnormal electrical activity in specific circumstances and elevating the risk of seizures^4^.

Nevertheless, there are studies suggesting that specific antidepressants may not impact the occurrence of seizures^5^. In fact, some research even suggests that certain antidepressants exhibit antiepileptic effects^6^. Current global guidelines assert that Selective Serotonin Reuptake Inhibitors (SSRIs) and Serotonin-Norepinephrine Reuptake Inhibitors (SNRIs) are recommended as the initial treatment for depression in individuals with epilepsy^7^^,^^8^. However, it is crucial to note that the level of evidence supporting these recommendations is relatively low, and the quality of the studies is not optimal. A systematic review identified the risk with amitriptyline, venlafaxine, trazodone, mirtazapine, paroxetine, and escitalopram among the antidepressants. For fluoxetine and duloxetine, the risk appears negligible^9^. However, another meta-analysis on the risk of antidepressants inducing seizures showed that users of newer-generation antidepressants have an increased risk of seizures, with short-term users exhibiting a more significant risk^10^.

Therefore, to date, evidence regarding the risk of seizures associated with the use of antidepressant drugs remains inconclusive. This suggests the need for larger sample sizes to comprehensively assess rare but significant adverse events such as seizures.

Leveraging the freely accessible FAERS database for adverse drug reaction surveillance proves cost-effective. Despite this, its untapped potential in probing the epileptogenic effects of antidepressant drugs prompted this analysis. The authors examined all antidepressant drug-associated seizure reports within FAERS, delving deeper into cases involving seizures.

## Methods

### Study design and data sources

In this retrospective observational pharmacovigilance study, the authors conducted a disproportionality analysis utilizing individual case safety reports derived from the Food and Drug Administration's (FDA) Adverse Event Reporting System (FAERS). The study dataset was assembled from the FAERS database (accessible via https://open.fda.gov/data/faers/) from Q1 2004 to Q4 2023. FAERS is a global pharmacovigilance database containing de-identified adverse drug event reports, with mandatory submissions from manufacturers and voluntary reports from healthcare providers and consumers. Reports originated from the U.S, the EU, and Asia.

The present analysis encompassed three core data components: the Personal Information Record (DEMO), the Drug Use Record (DRUG), and the Adverse Event Record (REAC). To uphold data integrity and mitigate redundancy, duplicate reports were systematically identified and excluded based on their unique case identifiers, in strict adherence to the FAERS duplicate handling protocols. For this study, the authors followed the STROBE guidelines for observational studies.

### Definition of antidepressant drugs

For this analysis, antidepressant drugs were extracted from the drugs using ANTIDEPRESSANTS (N06A) as classified by the WHO Anatomical Therapeutic Chemical Classification System. 65 antidepressant drugs were identified as Primary Suspected (PS) drugs^11^.

### Definition of seizures

The REAC and INDI tables categorize diseases using Preferred Terms (PTs) from the ICH International Medical Dictionary for Regulatory Activities (MedDRA)^12^. The MedDRA Standard Query (SMQ) compiles terms related to specific medical conditions. In this study, the emphasis was on “Seizures (MedDRA Code:10,039,911)” from MedDRA version 25.0, comprising 84 PTs. After excluding events that were unequivocally not drug-induced, such as Post-stroke seizure (PT: 10,076,981) and Post-traumatic epilepsy (PT: 10,036,312), a total of 41 PTs were identified and designated as “seizures” for the purposes of this study.

The analysis excluded seizure reports linked to co-existing conditions. After filtering out these conditions, such as seizures (81 PTs), cerebral injuries (30 PTs), CNS vascular disorders (215 PTs), brain tumors (115 PTs), and central nervous system infections and inflammations (181 PTs), the signal intensity was reevaluated for potential false positives. If the signal became undetectable post-adjustment, it was deemed a false signal due to confounding factors. This refinement aimed to reduce the risk of misidentifying signals attributed to these factors.

### Statistical analysis

The study used two methods, Reporting Odds Ratio (ROR) and Information Component (IC), to detect signals of drug-associated seizures^13^. The dual-method approach leverages ROR's sensitivity for frequent events and IC's robustness for rare adverse events, enhancing detection reliability. A signal was considered present if both ROR and IC met certain criteria, which were a case number of at least 3 and a lower limit of the 95 % Confidence Interval (95 % CI) > 1 for ROR, and an IC > 0 and a lower limit of the 95 % CI > 0 for IC. This threshold balances signal detection sensitivity and statistical stability, avoiding the exclusion of rare events while minimizing false positives from single-case reports. Calculations were performed using Microsoft Excel 2021 and SPSS version 27.0.

## Results

### Overview of adverse drug events (ADEs) reports submitted for antidepressant drugs in the FAERS database

**Fig. 1:**
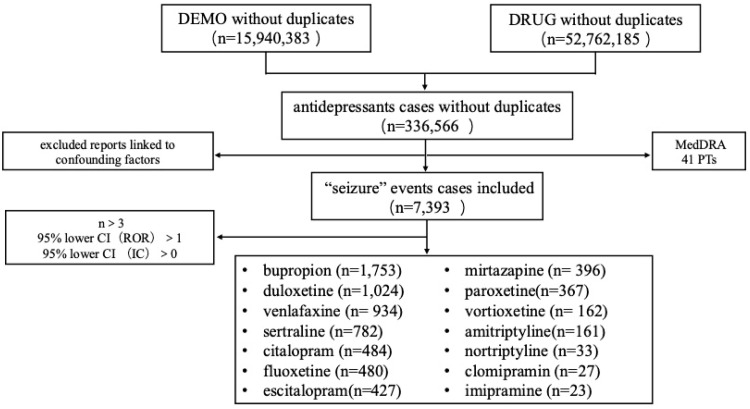
Flowchart of extraction and analysis of seizure-associated antidepressants from the FAERS database.

The process for extracting and analyzing antidepressants associated with seizures from the FAERS database is shown in Fig. 1. After applying inclusion and exclusion criteria and removing duplicate reports, a total of 15,940,383 ADE reports from 2004 to 2023 were obtained, including 336,566 reports of 65 target antidepressant drug-related adverse events, of which 7393 (1.29 %) were “seizure” events. The top 8 antidepressant drugs with the number of adverse event reports [the number of all event reports (the number of epileptic events reports, constituent ratio)] were: bupropion 25,939 (1757, 6.78 %), duloxetine 50,946 (1024, 2.01 %), venlafaxine 37,846 (934, 2.47 %), mirtazapine 16,159 (396, 2.45 %), vortioxetine 11,933 (162, 1.36 %), sertraline 46,492 (782, 1.68 %), citalopram 23,058 (484, 2.10 %), fluoxetine 22,600 (480, 2.12 %). An overview of the ADEs reports submitted for antidepressant drugs in the FAERS database is provided in Table 1.

**Table 1:** Basic information of the antidepressant-related seizure events.

		Case/n	%
Sex	Female	4028	54.5
	Male	2263	30.6
	Unknown	1102	14.9
	Total	7393	100
Age	<18	853	11.5
	≥65	799	10.8
	18‒65	3662	49.5
	Unknown	2079	28.1
	Total	7393	100
Report	HP	5172	70.0
	Non-HP	1933	26.1
	Unknown	288	3.9
	Total	7393	100
Region	Europe	1583	21.4
	North America	647	8.8
	Asian	169	2.3
	Oceania	24	0.3
	South America	8	0.1
	Africa	2	0.0
	Unknown	4960	67.1

HP, Healthcare Professionals.

The data indicates that the highest number of reported ADEs came from North America, Europe, and Asia. In terms of gender distribution, the data revealed that female patients (54.5 %) reported more ADEs than male patients (30.6 %). Additionally, patients who reported ADEs tended to be 18 to 65 years of age, accounting for 49.5 % of the total cases.

### Signal detection results

**Fig. 2:**
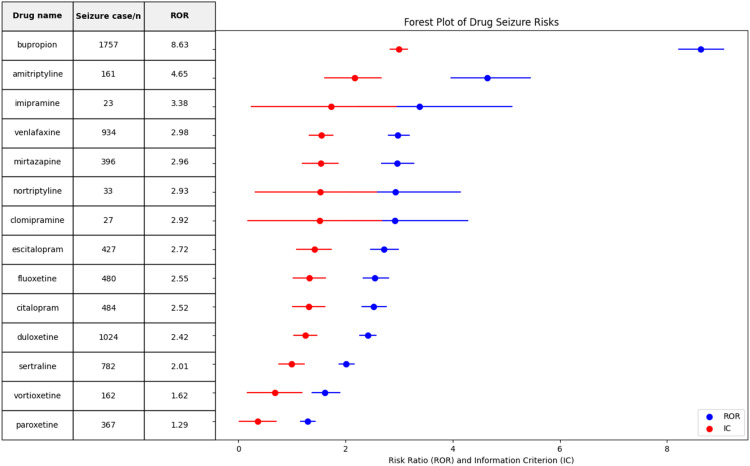
ROR and IC for seizures with antidepressants.

To reduce potential false signals caused by confounding factors, the authors adjusted for confounding factors. The complications that may lead to adverse events of epilepsy were excluded. The findings reveal a link between 14 out of 65 antidepressants and seizure events, as detailed in Fig. 2. Seizure RORs (95 % CI) for antidepressant drugs were, in descending order: bupropion 8.63 (8.22, 9.06), amitriptyline 4.65 (3.97, 5.45), imipramine 3.38 (2.23, 5.11), venlafaxine 2.98 (2.80, 3.19), mirtazapine 2.96 (2.67, 3.27), nortriptyline 2.93 (2.08, 4.14), clomipramine 2.92 (1.99, 4.28), escitalopram 2.72 (2.47, 2.99), fluoxetine 2.55 (2.33, 2.80), citalopram 2.52 (2.31, 2.76), duloxetine 2.42 (2.27, 2.57), sertraline 2.01 (1.88, 2.06), vortioxetine 1.62 (1.38, 1.89), paroxetine 1.29 (1.16, 1.43). IC outputs showed concordant signals without contradictions (full data in Supplement Table S1).

## Discussion

AE reports indicate that the incidence of adverse events is higher in women than in men (1.78:1). This could be related to the higher proportion of depression observed in women, leading to a greater number of patients taking antidepressants. Most patients are 18 to 65 years of age, consistent with the typical age of onset of conditions.

### Bupropion

Bupropion is a Norepinephrine/Dopamine reuptake inhibitor. The mechanism underlying seizures involves the increase of Norepinephrine (NE), which stimulates α_1_ adrenergic receptors. This excitation leads to heightened arousal and a reduction in the convulsion threshold, ultimately causing epileptic seizures. Numerous studies highlight bupropion's clear epileptogenicity. The risk of seizures is dose-dependent and varies with the dosage form. The use of bupropion in its Immediate Release (IR) form at dosages exceeding 450 mg can lead to a tenfold increase in seizure incidence^14^. However, the development of the Sustained Release (SR) formulation has significantly reduced this risk. In a study involving 3094 patients taking doses between 50 and 300 mg^15^, the incidence of seizures was reduced to 0.1 %. This rate is comparable to that of the general population (0.07 %‒0.09 %) and similar to other antidepressants, including SSRIs, which also have an incidence rate of 0.1 %^16^. However, in two recent systematic reviews, one did not find any risk of seizures associated with bupropion^10^, and the other found bupropion to have a relatively low correlation with seizures, ranking it 10th^9^. These findings contradict earlier studies.

Bupropion is contraindicated in epilepsy based on the drug label, which aligns with the ROR findings 8.63 (95 % CI 8.22‒9.06). In the present study, it exhibits the strongest signal. However, due to the lack of data, the authors cannot determine whether the patients were taking the IR or SR formulation. Consequently, the authors are unable to distinguish which dosage form is responsible for this signal.

### TCAs and tetracyclic antidepressants

According to current research findings, the incidence of seizures caused by TCA is approximately 1 %^17^. Maprotiline is a tetracyclic antidepressant associated with a relatively higher incidence of seizures. Reported data indicate that the incidence of maprotiline in clinical trials was initially 0.4 %, but subsequent post-marketing data show a much higher risk, with a strong dose relationship, leading to a reduction in its therapeutic dose range^18^^,^^19^. According to the meta-analysis in 2018^9^, clomipramine is the antidepressant with the highest risk of inducing seizures. The present study’s findings further establish the correlation between TCA antidepressants and the occurrence of seizures. In the present study, amitriptyline, imipramine, nortriptyline, and clomipramine exhibit strong correlations. Although TCAs and tetracyclic antidepressants are now less commonly used in patients with depression due to their side effects, the potential risk of seizures should still be acknowledged when using these medications.

### SSRI and SNRI

In this study, the authors found that, with the exception of fluvoxamine, other commonly used SSRIs and SNRIs are associated with a notable correlation to seizures. Venlafaxine exhibits the strongest signal, followed closely by escitalopram, sertraline, paroxetine, duloxetine, and trazodone.

SSRIs and SNRIs are generally considered to have a relatively low risk of inducing seizures among antidepressants. Despite the incomplete nature of current research, international medical guidelines still recommend SSRIs and SNRIs as the preferred treatment for depression in patients with epilepsy. This preference is due to their superior pharmacokinetic properties compared to MAOIs and TCAs, minimal drug interactions, and overall higher safety profile. However, it is important to note that the risk of seizures may increase with long-term treatment, high dosages, or overdose. The risk of seizures induced by SSRIs is approximately 0.1 %, which is relatively consistent across different drugs^16^. SNRI drugs such as venlafaxine and duloxetine have been shown in clinical trials to have seizure rates of approximately 0.3 % and 0.2 %, respectively.

Contrary to these findings, a recent meta-analysis^10^ indicated a significant association between the use of SSRIs/SNRIs (rather than TCAs) and the risk of seizures, particularly among short-term users.

Mechanistically, enhanced serotonin transmission can lower the epileptic threshold by concurrently activating 5-HT_1A_ and 5-HT_2_ receptors in the brainstem, potentially triggering myoclonus or epileptic seizures. Prolonged use of SSRIs can upregulate these receptors in the brainstem and inferior olivary nucleus, thereby modulating olivocerebellar rhythmicity, which could ultimately precipitate myoclonus or epileptic seizures.

The present study provides new evidence for the relationship between SSRIs/SNRIs and seizures, and highlights the need for careful consideration and monitoring of seizure risk in patients treated with SSRIs and SNRIs, especially in those with predisposing factors or when using higher doses over extended periods.

### Mirtazapine

The present study has detected a correlation between mirtazapine and seizures. Mirtazapine is a presynaptic α2-adrenergic antagonist that acts by promoting the release of norepinephrine and serotonin. In pre-marketing clinical trials involving 2796 patients, only 1 case of seizure was reported (0.04 %)^16^, leading to the belief that its risk of inducing seizures is low. However, subsequent post-marketing reports^20^^,^^21^ have shown that even patients without a predisposition to seizures may experience seizure events. Therefore, although the risk of seizure events with mirtazapine is relatively low, caution should still be exercised when using it in patients with epilepsy.

Although this study excluded individuals with pre-existing epilepsy or high-risk factors for seizures, similar to most clinical studies assessing the seizure risk of antidepressants, it still provides meaningful insights for clinical practice. By evaluating the seizure risk associated with various antidepressants in the general population, these findings may serve as an important reference when selecting antidepressants for patients with epilepsy comorbid with depression. Despite differences in the study population, the safety profiles identified here can inform risk-benefit considerations in clinical decision-making for this special group.

## Limitation

One important limitation of this analysis is the potential for underreporting of adverse events in the data source, which is derived from voluntarily submitted reports in the FAERS. This can lead to an incomplete understanding of the true frequency and severity of adverse drug reactions. Additionally, as with any observational study, it is difficult to establish a causal relationship between a drug and an adverse event due to the presence of confounding factors such as underlying health conditions and concomitant medications. In addition, the lack of data on dose, formulation, treatment duration, and substantial missing geographical information precludes analysis of dose-dependent risks, formulation differences, or regional variations. Furthermore, depression itself is one of the risk factors for epilepsy^22^, which adds complexity to the analysis. Patients with depression are more likely to experience seizures, possibly due to physiological and psychological changes associated with depression. The interplay between depression and epilepsy makes it difficult to distinguish adverse events caused by the drug from those resulting from the progression of the underlying disease or other treatments. In this FAERS study, the inability to completely isolate depression as a risk factor introduces a limitation. Despite these limitations, the large sample size of FAERS data can still provide valuable insights into new and rare drug-adverse event relationships.

## Conclusion

This study provides robust evidence supporting the association between 14 classes of antidepressant drugs and the incidence of seizures, utilizing data from FAERS. Through the evaluation of ROR for each class of antidepressants, the study elucidates the strength and significance of the association between different antidepressants and seizure events. These findings enhance the current understanding of antidepressant-related seizure risk and underscore the critical need for vigilant monitoring and risk assessment in patients undergoing antidepressant therapy. While these findings may inform antidepressant selection in patients with epilepsy, such applications require validation in dedicated studies due to distinct risk mechanisms in comorbid populations.

## Ethical and legal aspects

This study utilized publicly available data from the FAERS database. As the data are publicly available and de-identified, this study did not require approval from an ethics committee. However, the data were used in accordance with the terms and conditions of the FAERS database. This study utilized publicly available, de-identified data from the FAERS database. As the data are anonymized and publicly available, informed consent from individual patients was not required.

## Funding

This study was partially supported by a grant from the 10.13039/501100018542Natural Science Foundation of Sichuan Province (2023NSFSC1696).

## CRediT authorship contribution statement

**Dan Zou:** Conceptualization, Methodology, Software, Data curation, Writing – original draft. **Qiaozhi Hu:** Methodology, Software. **Lei Yu:** Supervision. **Bin Wu:** Software, Validation, Methodology, Data curation, Writing – review & editing.

## Declaration of competing interest

The authors declare that the research was conducted in the absence of any commercial or financial relationships that could be construed as a potential conflict of interest.

## Appendix Supplementary materials

Image, application 1
