# Supplementary material for: Analysis of the relationship between antidepressants and seizures based on the food and drug administration's adverse event reporting system database
Source: Clinics (Sao Paulo). 2025 Jul 15;80:100727. doi: 10.1016/j.clinsp.2025.100727 (PMC12281246; doi:10.1016/j.clinsp.2025.100727)
Supplement: Supplementary file 1 [file mmc1.docx]

CLINICS-D-25-00231_Supplemnetary Material

**Table S1** ROR and IC for seizures with antidepressants.

| **Group** | **Drug/PT** | **Seizure case** | **b** | **c** | **d** | **ROR** | **95% CI lower** | **95% CI upper** | **IC** | ***95% CI lower*** | ***95% CI upper*** |
| --- | --- | --- | --- | --- | --- | --- | --- | --- | --- | --- | --- |
| Antidepressants | Antidepressants | 7393 | 329173 | 127214 | 15,476,603 | **2.73** | 2.67 | 2.80 | **1.38** | 1.30 | 1.46 |
| OA | OA | 4539 | 174832 | 130068 | 15,630,944 | **3.12** | 3.03 | 3.21 | **1.58** | 1.48 | 1.68 |
| SSRi | SSRi | 2563 | 143418 | 132044 | 15,662,358 | **2.12** | 2.04 | 2.21 | **1.06** | 0.92 | 1.19 |
| non-SMRi | Non-smri | 282 | 9486 | 134325 | 15,796,290 | **3.50** | 3.11 | 3.94 | **1.77** | 1.37 | 2.15 |
| MOi | Moi | 9 | 1437 | 134598 | 15,804,339 | **0.74** | 0.38 | 1.42 | **-0.44** | -2.47 | 1.66 |
| OA | Bupropion | 1757 | 24182 | 132850 | 15,781,594 | **8.63** | 8.22 | 9.06 | **3.00** | 2.84 | 3.16 |
| OA | Duloxetine | 1024 | 49922 | 133583 | 15,755,854 | **2.42** | 2.27 | 2.57 | **1.25** | 1.04 | 1.46 |
| OA | Venlafaxine | 934 | 36912 | 133673 | 15,768,864 | **2.98** | 2.80 | 3.19 | **1.55** | 1.33 | 1.76 |
| OA | Mirtazapine | 396 | 15763 | 134211 | 15,790,013 | **2.96** | 2.67 | 3.27 | **1.54** | 1.20 | 1.86 |
| OA | Vortioxetine | 162 | 11771 | 134445 | 15,794,005 | **1.62** | 1.38 | 1.89 | **0.68** | 0.17 | 1.19 |
| OA | Desvenlafaxine | 78 | 17303 | 134529 | 15,788,473 | **0.53** | 0.42 | 0.66 | **-0.91** | -1.64 | -0.17 |
| OA | Vilazodone | 59 | 5129 | 134548 | 15,800,647 | **1.35** | 1.05 | 1.75 | **0.43** | -0.42 | 1.27 |
| OA | Trazodone | 58 | 4552 | 134549 | 15,801,224 | **1.50** | 1.15 | 1.94 | **0.58** | -0.29 | 1.42 |
| OA | Esketamine | 48 | 4580 | 134559 | 15,801,196 | **1.23** | 0.93 | 1.64 | **0.30** | -0.65 | 1.22 |
| OA | Milnacipran | 7 | 2645 | 134600 | 15,803,131 | **0.31** | 0.15 | 0.65 | **-1.68** | -3.85 | 0.76 |
| OA | Viloxazine | 6 | 245 | 134601 | 15,805,531 | **2.88** | 1.28 | 6.46 | **1.50** | -1.33 | 3.66 |
| OA | Levomilnacipran | 5 | 668 | 134602 | 15,805,108 | **0.88** | 0.36 | 2.12 | **-0.18** | -2.83 | 2.51 |
| OA | Nefazodone | 3 | 1091 | 134604 | 15,804,685 | **0.32** | 0.10 | 1.00 | **-1.62** | -4.62 | 1.90 |
| OA | Tianeptine | 2 | 24 | 134605 | 15,805,752 | **9.79** | 2.31 | 41.41 | **3.19** | -2.67 | 5.25 |
| OA | Agomelatine | 0 | 11 | 134607 | 15,805,765 | **0.00** | #NUM! | #NUM! | **#NUM!** | -6.92 | 6.64 |
| OA | Mianserin | 0 | 10 | 134607 | 15,805,766 | **0.00** | #NUM! | #NUM! | **#NUM!** | -6.93 | 6.67 |
| OA | Reboxetine | 0 | 13 | 134607 | 15,805,763 | **0.00** | #NUM! | #NUM! | **#NUM!** | -6.90 | 6.58 |
| OA | Tryptophan | 0 | 11 | 134607 | 15,805,765 | **0.00** | #NUM! | #NUM! | **#NUM!** | -6.92 | 6.64 |
| SSRi | Sertraline | 782 | 45710 | 133825 | 15,760,066 | **2.01** | 1.88 | 2.16 | **0.99** | 0.76 | 1.23 |
| SSRi | Citalopram | 484 | 22574 | 134123 | 15,783,202 | **2.52** | 2.31 | 2.76 | **1.31** | 1.01 | 1.61 |
| SSRi | Fluoxetine | 480 | 22120 | 134127 | 15,783,656 | **2.55** | 2.33 | 2.80 | **1.33** | 1.03 | 1.63 |
| SSRi | Escitalopram | 427 | 18497 | 134180 | 15,787,279 | **2.72** | 2.47 | 2.99 | **1.42** | 1.09 | 1.73 |
| SSRi | Paroxetine | 367 | 33448 | 134240 | 15,772,328 | **1.29** | 1.16 | 1.43 | **0.36** | 0.02 | 0.70 |
| SSRi | Fluvoxamine | 23 | 1069 | 134584 | 15,804,707 | **2.53** | 1.67 | 3.82 | **1.32** | -0.11 | 2.57 |
| non-SMRi | Amitriptyline | 161 | 4068 | 134446 | 15,801,708 | **4.65** | 3.97 | 5.45 | **2.17** | 1.62 | 2.66 |
| non-SMRi | Nortriptyline | 33 | 1321 | 134574 | 15,804,455 | **2.93** | 2.08 | 4.14 | **1.53** | 0.32 | 2.58 |
| non-SMRi | Clomipramine | 27 | 1086 | 134580 | 15,804,690 | **2.92** | 1.99 | 4.28 | **1.52** | 0.18 | 2.67 |
| non-SMRi | Doxepin | 27 | 1764 | 134580 | 15,804,012 | **1.80** | 1.23 | 2.63 | **0.84** | -0.44 | 2.04 |
| non-SMRi | Imipramine | 23 | 800 | 134584 | 15,804,976 | **3.38** | 2.23 | 5.11 | **1.73** | 0.24 | 2.94 |
| non-SMRi | Desipramine | 6 | 160 | 134601 | 15,805,616 | **4.40** | 1.95 | 9.95 | **2.10** | -0.97 | 4.05 |
| non-SMRi | Maprotiline | 4 | 163 | 134603 | 15,805,613 | **2.88** | 1.07 | 7.77 | **1.50** | -1.91 | 4.00 |
| non-SMRi | Trimipramine | 1 | 49 | 134606 | 15,805,727 | **2.40** | 0.33 | 17.35 | **1.24** | -4.21 | 5.18 |
| non-SMRi | Amoxapine | 0 | 28 | 134607 | 15,805,748 | **0.00** | #NUM! | #NUM! | **#NUM!** | -6.94 | 6.31 |
| non-SMRi | Dosulepin | 0 | 10 | 134607 | 15,805,766 | **0.00** | #NUM! | #NUM! | **#NUM!** | -6.93 | 6.67 |
| non-SMRi | Lofepramine | 0 | 1 | 134607 | 15,805,775 | **0.00** | #NUM! | #NUM! | **#NUM!** | -8.00 | 7.95 |
| non-SMRi | Opipramol | 0 | 11 | 134607 | 15,805,765 | **0.00** | #NUM! | #NUM! | **#NUM!** | -6.92 | 6.64 |
| non-SMRi | Protriptyline | 0 | 25 | 134607 | 15,805,751 | **0.00** | #NUM! | #NUM! | **#NUM!** | -6.92 | 6.35 |
| MOi | Phenelzine | 6 | 1025 | 134601 | 15,804,751 | **0.69** | 0.31 | 1.53 | **-0.54** | -2.94 | 2.00 |
| MOi | Tranylcypromine | 2 | 390 | 134605 | 15,805,386 | **0.60** | 0.15 | 2.42 | **-0.73** | -4.30 | 3.25 |
| MOi | Moclobemide | 1 | 9 | 134606 | 15,805,767 | **13.05** | 1.65 | 102.98 | **3.57** | -4.13 | 5.88 |
| MOi | Isocarboxazid | 0 | 13 | 134607 | 15,805,763 | **0.00** | #NUM! | #NUM! | **#NUM!** | -6.90 | 6.58 |
